# Genome rearrangements and megaplasmid loss in the filamentous bacterium *Kitasatospora viridifaciens* are associated with protoplast formation and regeneration

**DOI:** 10.1007/s10482-020-01393-7

**Published:** 2020-02-14

**Authors:** Karina Ramijan, Zheren Zhang, Gilles P. van Wezel, Dennis Claessen

**Affiliations:** Molecular Biotechnology, Institute of Biology, Leiden University, P.O. Box 9505, 2300 RA Leiden, The Netherlands

**Keywords:** Genetic instability, Actinobacteria, Protoplast, Insertion sequence, Heterogeneity

## Abstract

Filamentous Actinobacteria are multicellular bacteria with linear replicons. *Kitasatospora viridifaciens* DSM 40239 contains a linear 7.8 Mb chromosome and an autonomously replicating plasmid KVP1 of 1.7 Mb. Here we show that lysozyme-induced protoplast formation of the multinucleated mycelium of *K. viridifaciens* drives morphological diversity. Characterisation and sequencing of an individual revertant colony that had lost the ability to differentiate revealed that the strain had not only lost most of KVP1 but also carried deletions in the right arm of the chromosome. Strikingly, the deletion sites were preceded by insertion sequence elements, suggesting that the rearrangements may have been caused by replicative transposition and homologous recombination between both replicons. These data indicate that protoplast formation is a stressful process that can lead to profound genetic changes.

**Electronic supplementary material:**

The online version of this article (10.1007/s10482-020-01393-7) contains supplementary material, which is available to authorized users.

## Introduction

Filamentous Actinobacteria are prolific producers of bioactive compounds. These metabolites are mostly used as weapons that provide protection against other microorganisms and phages in the environment (Abrudan et al. 2015; Kronheim et al. 2018; van der Heul et al. 2018). This is particularly useful for filamentous organisms, given that they generally lack the ability to make flagella for escaping dangerous situations. In addition, these bacteria are able to generate resistant spores that can invade new environments after their dispersal. Germination of spores leads to the formation of 1–2 germ tubes, which grow by tip extension, thereby establishing filamentous cells called hyphae. Branching of hyphae leads to the formation of a multinucleated vegetative mycelium, which forages and acquires nutrients by decomposing polymeric substances. Stressful conditions (such as nutrient depletion) induce program cell death (PCD) of the mycelium, which in turn triggers morphological and chemical differentiation (Claessen et al. 2014). This developmental transition leads to the formation of specialised hyphae that grow into the air, and the onset of production of a suite of bioactive compounds (Barka et al. 2016). Eventually, the aerial hyphae metamorphose into chains of grey-pigmented spores. Mutants that are unable to establish an aerial mycelium are called bald (*bld*), while those that are not capable to form spores are called white (*whi*) after their whitish color (Chater 2001; Claessen et al. 2006).

Genome mining has been instrumental for the revival of drug discovery (Bentley et al. 2002; Ichikawa et al. 2010). Many of the biosynthetic gene clusters that specify bioactive natural products are contained on giant linear plasmids (Chater and Kinashi 2007; Kinashi 2011; Medema et al. 2010; Mochizuki et al. 2003). Although linear replicons are rare in many bacterial taxa, they are common in Actinobacteria (Chen 1996; Kirby and Chen 2011). In fact, *Streptomyces* chromosomes (between 8 and 10 Mb in size) are also linear and typically comprise a “core region” containing the essential genes, and two variable “arms” with lengths ranging from 1.5 to 2.3 Mb (Hopwood 2006). Like linear plasmids, the linear chromosomes are capped by terminal proteins bound to the 5′ end of the DNA (Nindita et al. 2015). The chromosomal ends are genetically unstable, and readily undergo large (up to 2 Mb) DNA rearrangements. Such rearrangements can lead to circularization of the chromosome, exchange of chromosomal arms or the formation of hybrid chromosomes due to recombination between the linear plasmids and the chromosome (Hoff et al. 2018). This wide range of genomic rearrangements is believed to be caused by transposition or homologous recombination, occurring actively within the chromosome or between the chromosome and linear plasmids (Chen et al. 2002). Not surprisingly, these changes have profound effects on differentiation and specialised metabolite production (Leblond and Decaris 1994; Zhang et al. 2020).

Here we characterized genetic instability in *Kitasatospora viridifaciens.* This tetracycline producer was originally classified within the genus *Streptomyces*, it was recently shown to belong to the genus *Kitasatospora* (Girard et al. 2014). Protoplast formation and regeneration leads to the emergence of colonies that are no longer able to differentiate, which we attribute to the deletion of a 1.5 Mb segment of the right chromosomal arm and concomitant loss of most of the sequences contained on the large megaplasmid KVP1.

## Methods

### Strains and media

The strains used in this study (Table 1) are derivatives of *K. viridifaciens* DSM40239 (DSMZ). For protoplast preparation a spore suspension (10^6^ spores ml^−1^) was grown for 48 h in a mixture of TSBS-YEME (1:1 v/v) supplemented with 5 mM MgCl_2_ and 0.5% glycine. Protoplasts were prepared as described (Kieser et al. 2000), with the difference that the lysozyme concentration was increased to 10 mg ml^−1^. Serial dilutions of protoplasts were plated on R5 (Kieser et al. 2000) or MYM medium (Stuttard 1982) at 30 °C. Regenerated protoplasts were streaked twice to single colonies on MYM before selecting the three independent bald colonies (B3.1, B3.2, and B3.3) that were further analyzed. Bald colonies were used as inoculum on liquid cultures of TSBS. Genomic DNA was isolated after 2 days of growth at 30 °C.

**Table 1:** Strains used in this study

Strains	Characteristics	Genotype	Reference
DSM 40239	Sporulating parent strain	Wild-type *K. viridifaciens*	DSMZ
G1-G3	Sporulating revertant	N.D.	This work
B1	Revertant that recovered the ability to sporulate in the second subculture	N.D.	This work
B2	Revertant with a bald colony morphology	N.D.	This work
B3	Revertant with a mixed colony morphology	N.D.	This work
B3.1	Revertant with a bald colony morphology	KVP1 minus, 1 Mb deletion in right arm of the chromosome	This work
B3.2	Revertant with a bald colony morphology	KVP1 minus	This work
B3.3	Revertant with a bald colony morphology	KVP1 minus	This work

*N.D.* Not determined

### Whole genome sequencing and analysis

For genomic DNA isolation strains were grown in Tryptic Soy Broth medium containing 10% sucrose until mid-exponential phase. Next, chromosomal DNA was isolated as described previously (Kieser et al. 2000) and sequenced by BaseClear (Leiden, The Netherlands). Alignments of Illumina reads were performed using CLC Genomics Workbench 8.5.1. Raw Illumina (Hiseq 2500 system) sequences of the bald strain B3.1 were imported and mapped to the reference genome of *K. viridifaciens* DSM40239 (NCBI reference sequence: NZ_MPLE00000000.1) through the “Map reads to reference” function in the NGS core tools. Mismatch cost was set to 2 and non-specific matches were handled by mapping them randomly.

### Pulsed-field gel electrophoresis

For Pulsed-Field Gel Electrophoresis (PFGE), 10^6^ spores ml^−1^ of *K. viridifaciens* were inoculated in 25 ml of TSBS with 0.5% glycine or LPB (Ramijan et al. 2018). Cultures were grown at 30 °C agitating at 200 rpm for 16 and 40 h, respectively. Mycelial pellets were harvested by centrifugation at 4000 rpm for 15 min. The preparation of plugs for PFGE was performed as previously described (Zhang et al. 2020). Plugs were made with SeaKem Gold agarose (Lonza, Switzerland), and the genomic DNA in the plugs was cut with AseI. Plugs were run using a CHEF-DR II PFGE system (Biorad, USA). For efficient separation of fragments, samples were run in two conditions: a switching time of 60–125 s for 20 h, or a switching time of 2.2–75 s for 19 h, both at 200 V.

### Quantitative real time PCR

Aliquots of 5 ng of DNA were used as a template in quantitative real time PCR. We used primers for both chromosomal (*atpD* and *infB*) and KVP1 (*allC*, *tetR*, *parA* and *orf1*) genes (Table 2). The PCR reactions were performed with the iTaq Universal SYBR Green Supermix Mix (Bio-Rad) using 5% DMSO, according to the manufacturer’s instructions. Reactions was performed in duplicate using a CFX96 Touch Real-Time PCR Detection System (Bio-Rad). To normalise the relative amount of DNA, the wild-type strain was used as a control, using the *atpD* gene as a reference.

**Table 2:** Primers used for quantitative real time PCR

Primer	Sequence (5′–3′)
qPCR_*infB*-Fw	GTCACGTCGACCACGGTAAG
qPCR_*infB*-Rv	CACCGATGTGCTGGGTGATG
qPCR_*atpD*-Fw	TTCGGACAGCTCGTCCATAC
qPCR_*atpD*-Rv	ACATCGCGCAGAACCACTAC
qPCR_*parA*-Fw	CGGTCGTCACCCAGTACAAG
qPCR-*parA*-Rv	TAACCGAGTTCGAGGGACAG
qPCR-o*rf1*-Fw	GAGGGAGCCAATCCCGTATC
qPCR-o*rf1*-Rv	GGCTGTTGGACAGGACCATC
qPCR-*allC*-Fw	CGGCGATAGCGGAGACTAAG
qPCR-*allC*-Rv	CCACTGGTGGGACCAGAAAG
qPCR-*tetR*-Fw	TGCTCGACCAGCTGTTGAAG
qPCR-*tetR*-Rv	TGGCGAGCATGAAGTCGTAG

## Results

### Genomic characterisation of *Kitasatospora viridifaciens*

**Fig. 1:**
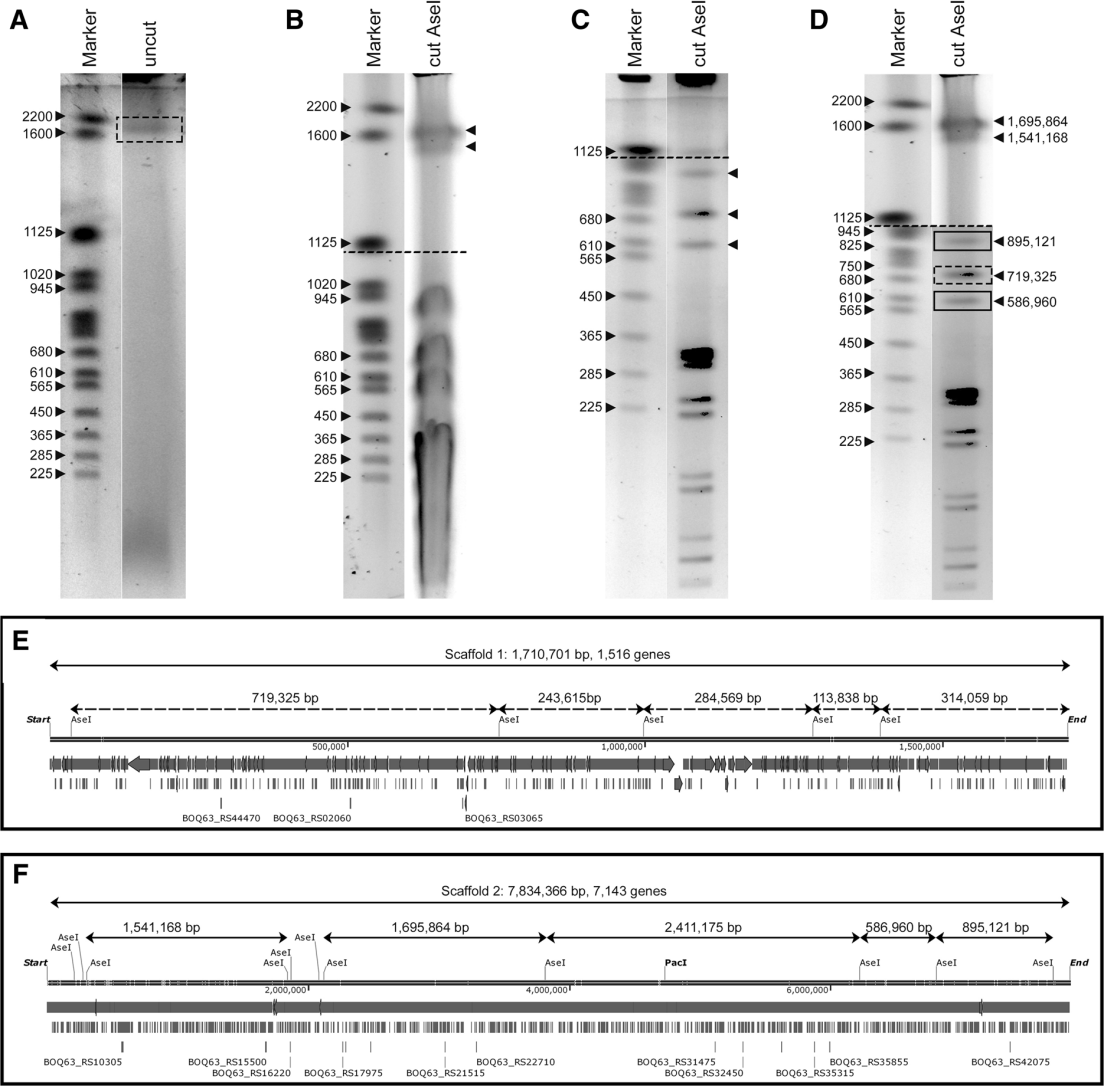
KVP1 of *K. viridifaciens* is a megaplasmid. Pulsed-Field Gel Electrophoresis of genomic DNA of *K. viridifaciens* grown in TSBS (**a**, **c**) or LPB (**b**) medium. DNA was separated using switching times of 60–125 s for 20 h (**a**, **b**) or 2.2–75 s for 19 h (**c**). **d** The composite gel shows fragments larger than 1,125,000 bp (derived from the gel shown in panel **b**), and fragments smaller than 1,020,000 bp (derived from panel **c**). The solid rectangles indicate fragments derived from the chromosome, while the dashed rectangles indicate fragments derived from KVP1. Predicted in silico maps of the KVP1 megaplasmid and chromosome of *K. viridifaciens* are shown in panels (**e**) and (**f**), respectively

We previously sequenced *K. viridifaciens* and identified KVP1 as a novel megaplasmid (Ramijan et al. 2017). Analysis of the biosynthetic gene clusters (BGCs) using antiSMASH 5.0 (Blin et al. 2019) located 11 BGCs on KVP1 and 33 clusters on the chromosome (Fig. S1). One of the BGCs showed high homology to the BGC for chlortetracycline (Fig. S2). To test experimentally whether KVP1 is indeed a plasmid, we analysed genomic DNA of the wild-type strain with Pulsed-Field Gel Electrophoresis (PFGE). In the lane containing uncut DNA, a fragment with an estimated size between 1,600,000 and 2,200,000 bp (Fig. 1a, boxed region) was evident. This fragment is consistent with a genetic element that migrates independently of the chromosomal DNA. Digestion of the DNA with AseI revealed multiple DNA fragments, including two large fragments at 1,541,168 and 1,695,864 bp (see arrowheads in Fig. 1b). By further adjusting the switching time to 2.2–75 s, well-separated fragments with sizes ranging from 565,000 to 945,000 bp were identified (arrowheads Fig. 1c). Combining these different PFGE runs (Fig. 1d) allowed us to map the AseI fragment spectrum to the in silico genome assembly of *K. viridifaciens* (Fig. 1e–f). Altogether, these results confirmed that KVP1 is a megaplasmid and verified the predicted AseI sites in the chromosome.

### Protoplast formation and regeneration leads to morphological diversity due to deletions and rearrangements in the chromosome and KVP1

**Fig. 2:**
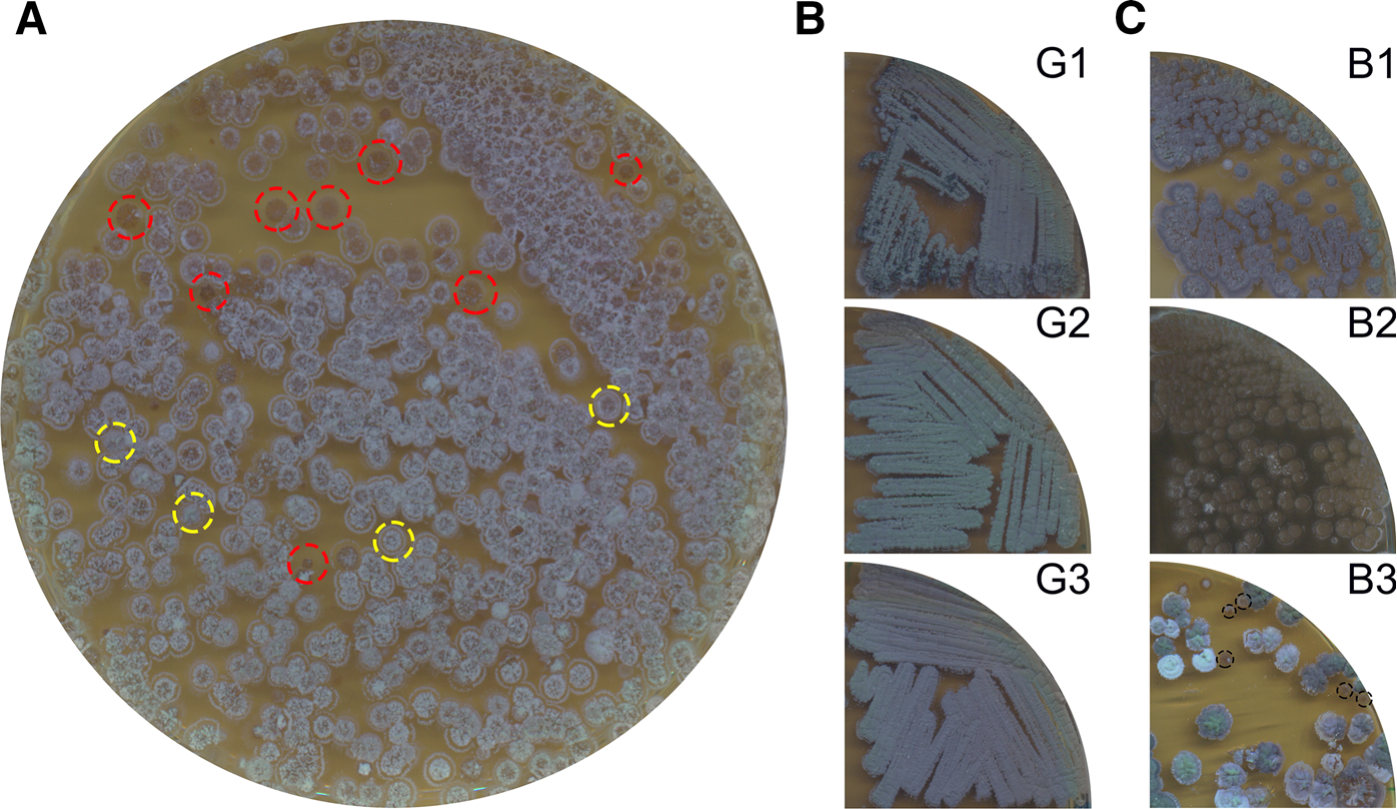
Protoplast formation and regeneration yield colonies with developmental defects (**a**) Protoplasts of *K. viridifaciens* regenerated on MYM medium yields grey-pigmented colonies (yellow dotted circles) and brown colonies (red dotted circles). The grey colonies retain their morphology in subsequent subcultures (**b**). **c** Subculturing of the brown colonies reveals morphological heterogeneity: some colonies appear grey-pigmented (B1), while others are bald (B2) or display a variety of phenotypes (B3). Bald colonies in B3 are indicated with black dashed circles

The identification of KVP1 as a megaplasmid prompted us to analyse if it was distributed homogenously throughout the mycelium. For this, protoplasts were generated using a standard lysozyme-based protocol. Surprisingly, when protoplasts were allowed to regenerate on MYM agar plates, many colonies had developmental defects. Although the majority of colonies formed grey-pigmented spores (yellow circles Fig. 2a), a significant number of colonies was brown and failed to develop (red circles Fig. 2a). Sub-culturing of these so-called bald colonies (referred to as “b” in Fig. 2c) resulted in three morphological phenotypes, namely grey-pigmented colonies (B1), bald colonies (B2) or colonies with a variety of phenotypes (B3).

To rule out that the MYM medium, which lacks osmoprotectant agents, was the main cause of the morphological differences, protoplasts were also allowed to regenerate on the more commonly used R5 medium (Kieser et al. 2000). None of the colonies that arose after regeneration of protoplasts were able to differentiate on R5 medium, which is typical of *K. viridifaciens* (Fig. S3A). To analyse this further, 149 colonies were randomly selected from R5 agar plates and subsequently streaked onto MYM agar (Fig. S3B). After 7 days of growth, 77% of the colonies had an apparent wild-type morphology and produced grey-pigmented spores, while 23% of the colonies were defective in development. This demonstrates that the observed morphologically heterogeneity detected after protoplast formation and regeneration is medium-independent.

**Fig. 3:**
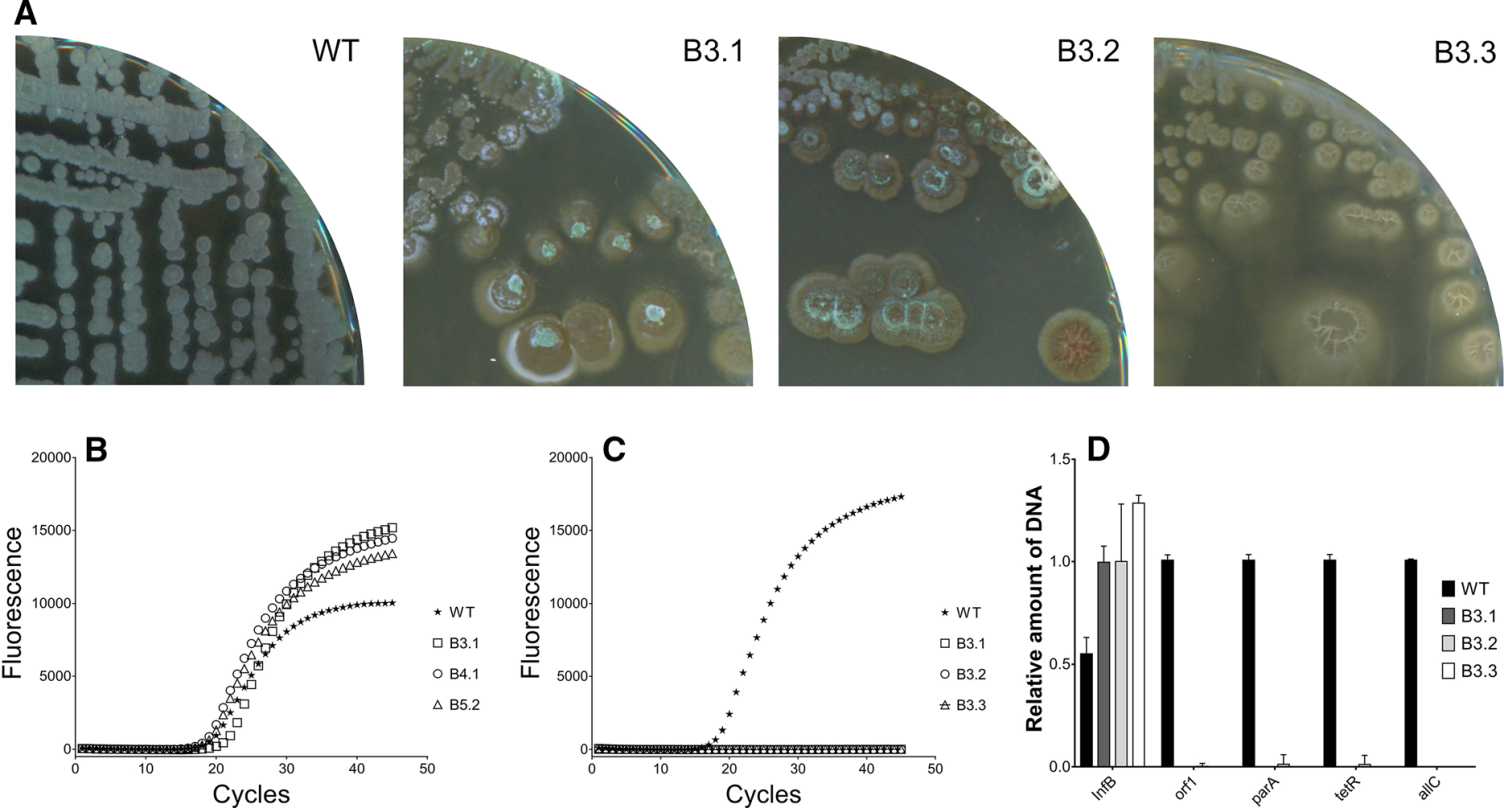
Bald colonies have lost the KVP1 megaplasmid. **a** Three independently isolated bald strains (B3.1, B3.2 and B3.3) are unable to sporulate on MYM medium, unlike the wild-type strain. Quantitative real-time PCR showed the presence of chromosomal gene *infB* in both the wild-type and bald strains (**b**). In contrast, the *allC* gene located on KVP1 is only present in the wild-type strain (**c**). **d** The relative abundance of four megaplasmid genes (*orf1*, *parA*, *tetR*, *allC*) in comparison to the abundance of the chromosomal gene *infB* suggest that KVP1 is lost in strains B3.1, B3.2 and B3.3

The (partial) loss of megaplasmids is known to cause morphological defects similar to those observed here (Álvarez-Álvarez et al. 2014). To test if loss of KVP1 explains the change in phenotype, three B3-type bald colonies (encircled in Fig. 2c) were streaked onto MYM agar plates (Fig. 3a). All three colonies showed severe morphological defects and failed to produce spores even after 14 days of cultivation (Fig. 3a). Total DNA was then extracted from the three lineages (B3.1, B3.2, B3.3), and analysed for genes that served as markers for either the chromosome or for KVP1. Quantitative real-time PCR detected the chromosomal gene *infB* in the wild-type strain and the three tested lineages before the 20th cycle of amplification (Fig. 3b). Conversely, the *allC* gene located on the KVP1 plasmid was only detected in the wild-type strain (Fig. 3c). Similarly, we were unable to detect other KVP1-specific genes, namely *orf1, parA* or *tetR* (Fig. 3d). These results strongly suggested the loss of KVP1 in these colonies.

**Fig. 4:**
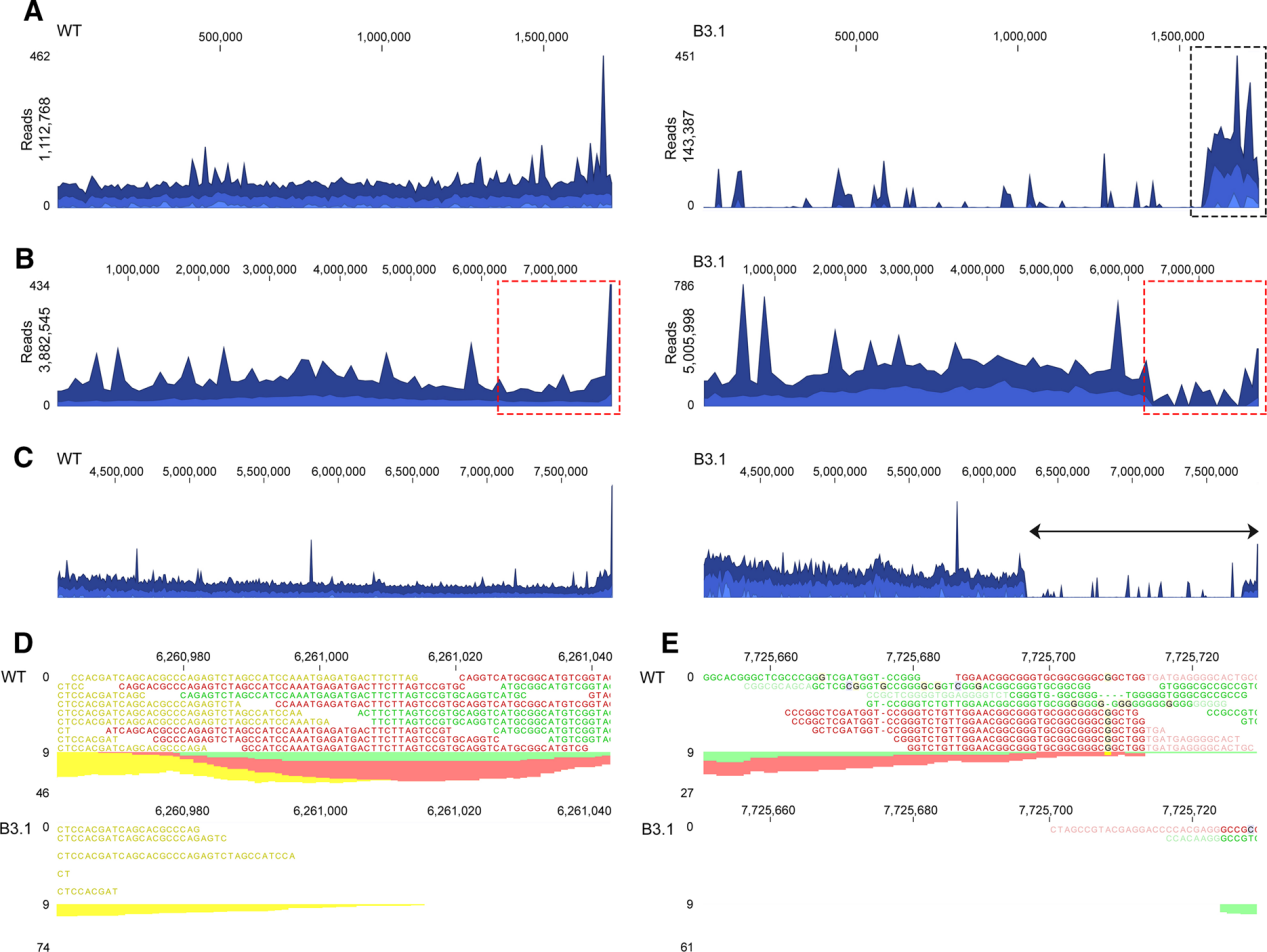
Whole genome sequencing reveals major chromosomal and megaplasmid deletions in strain B3.1. Alignment of Illumina reads of the wild type (left) and B3.1 strain (right) against KVP1 (**a**) and the chromosome (**b**). Please note the high coverage of KVP1 sequences (panel A) detected in the wild type (1,112,768 reads) compared to those of strain B3.1 (143,387 reads). Similarly, a high coverage in the right arm of the chromosome (dashed red rectangle) is observed for the wild-type (**b**, left panel) in comparison to strain B3.1 (**b**, right panel). **c**–**e** A more detailed characterisation reveals that all reads between 6,261,000 and 7,725,700 are absent in the right arm of the chromosome of strain B3.1

To corroborate the loss of KVP1, next-generation sequencing of the total DNA of strain B3.1 was performed. While the wild-type strain showed the expected distribution of reads over genome and plasmid (3,882,545 and 1,112,768 respectively), the number of reads mapping to KVP1 was dramatically underrepresented in B3.1 (Fig. 4a, b, right panels with 5,005,998 and 143,387 reads for the chromosome and KVP1, respectively). These KVP1-mapped reads corresponded to 164,769 bp of the plasmid, mostly located on the terminal end of the right arm (black box in Fig. 4a, right column). Interestingly, the number of reads mapping to the right arm of the chromosome was also dramatically decreased in B3.1 (Fig. 4b right column, dashed red rectangle). A more detailed analysis indicated that most chromosomal sequences between 6,261,000 and 7,725,700 bp (Fig. 4c, arrow) were absent in B3.1. Apparently, strain B3.1 had not only lost the majority of sequences contained on KVP1, but also a major part of its right chromosomal arm. Further investigation of the deletion sites revealed an insertion sequence (IS) immediately adjacent to the chromosomal deletion start (around 6,261,000 bp) in B3.1 (Table 3). This IS contains the BOQ63_RS37135 gene encoding the transposase likely involved in moving this element. Furthermore, close inspection of KVP1 sequences still present in strain B3.1 also identified a flanking IS element containing the BOQ63_RS06880 transposase (Table 4). Altogether, these results demonstrate that major chromosomal and megaplasmid rearrangements and DNA loss occur during protoplast formation and regeneration, which is likely mediated via transposition events.

**Table 3:** Transposition elements in the *K. viridifaciens* chromosome

Locus	Product	Location	
		Start	End
BOQ63_RS09605	IS5/IS1182 family transposase	408,874	409,442
BOQ63_RS10710	Transposase	656,353	657,240
BOQ63_RS10725	IS256 family transposase	659,679	660,950
BOQ63_RS45020	IS5/IS1182 family transposase	1,013,156	1,014,156
BOQ63_RS12740	IS110 family transposase	1,086,937	1,088,178
BOQ63_RS12750	IS5/IS1182 family transposase	1,088,200	1,088,903
BOQ63_RS15035	IS200/IS605 family transposase	1,569,116	1,569,363
BOQ63_RS15720	Transposase	1,761,237	1,761,554
BOQ63_RS15725	Transposase	1,761,551	1,762,465
BOQ63_RS17065	Transposase	2,044,875	2,046,032
BOQ63_RS20605	Transposase	2,847,519	2,848,817
BOQ63_RS25160	Transposase	3,812,365	3,813,189
BOQ63_RS25165	IS21 family transposase	3,813,189	3,814,457
BOQ63_RS25685	Transposase	3,933,744	3,934,664
BOQ63_RS25690	Transposase	3,934,661	3,934,972
BOQ63_RS26595	IS21 family transposase	4,116,603	4,117,871
BOQ63_RS26600	Transposase	4,117,871	4,118,695
BOQ63_RS26675	IS5/IS1182 family transposase	4,131,168	4,132,061
BOQ63_RS29050	Transposase	4,605,449	4,606,369
BOQ63_RS32225	IS630 family transposase	5,238,044	5,284,135
BOQ63_RS34385	Transposase	5,704,658	5,705,617
BOQ63_RS37135*	IS5/IS1182 family transposase	6,260,623	6,260,976
BOQ63_RS37400	IS110 family transposase	6,329,086	6,330,300
BOQ63_RS40925	Transposase	7,097,036	7,097,923
BOQ63_RS40980	IS5/IS1182 family transposase	7,106,937	7,107,098
BOQ63_RS41025	IS481 family transposase	7,118,421	7,118,739
BOQ63_RS41195	IS21 family transposase	7,174,951	7,176,219
BOQ63_RS41200	Transposase	7,176,219	7,177,037
BOQ63_RS41470	IS110 family transposase	7,239,487	7,240,701
BOQ63_RS42020	Transposase	7,367,798	7,369,021

*Transposase found upstream chromosomal right arm deletion in bald strain B3.1

**Table 4:** Transposition elements in the right terminal region of KVP1

Locus	Product	Location	
		Start	End
BOQ63_RS06800*	IS5/IS1182 family transposase	1,545,936	1,546,388
BOQ63_RS06805**	IS5/IS1182 family transposase	1,546,385	1,546,738
BOQ63_RS06815	Transposase	1,547,179	1,548,066
BOQ63_RS06880**	IS5/IS1182 family transposase	1,558,369	1,558,722
BOQ63_RS06915	Transposase	1,564,436	1,564,969
BOQ63_RS06940	IS5/IS1182 family transposase	1,598,415	1,569,308
BOQ63_RS07050**	IS5/IS1182 family transposase	1,586,628	1,586,981
BOQ63_RS07055	IS5/IS1182 family transposase	1,586,978	1,587,430
BOQ63_RS07060	IS5/IS1182 family transposase	1,587,553	1,588,391
BOQ63_RS07125	IS110 family transposase	1,600,675	1,600,800
BOQ63_RS07220	IS5/IS1182 family transposase	1,621,157	1,622,011
BOQ63_RS07225	IS5/IS1182 family transposase	1,622,341	1,623,179
BOQ63_RS07245	IS5/IS1182 family transposase	1,624,729	1,625,079
BOQ63_RS07255	IS110 family transposase	1,625,394	1,626,635
BOQ63_RS07260**	IS5/IS1182 family transposase	1,627,012	1,627,365
BOQ63_RS07280	IS5/IS1182 family transposase	1,630,283	1,630,534
BOQ63_RS07290	Transposase	1,631,323	1,632,114
BOQ63_RS07295	Transposase	1,632,285	1,633,493
BOQ63_RS07305	Site-specific integrase	1,634,154	1,635,464
BOQ63_RS07320	DNA invertase	1,636,936	1,637,601
BOQ63_RS07335	DDE transposase	1,640,507	1,643,533
BOQ63_RS07345	Integrase	1,645,074	1,646,333
BOQ63_RS07380	Integrase	1,652,931	1,654,022
BOQ63_RS07400	Resolvase	1,656,203	1,656,996
BOQ63_RS07425	Transposase	1,658,975	1,659,889
BOQ63_RS07430	DDE transposase	1,660,210	1,661,212
BOQ63_RS07440	Transposase	1,661,565	1,662,452
BOQ63_RS07445	IS30 family transposase	1,662,486	1,662,824
BOQ63_RS07475	IS21 family transposase	1,665,953	1,667,221
BOQ63_RS07480	IS21 family transposase	1,667,436	1,668,701
BOQ63_RS07485	Transposase	1,668,701	1,669,546
BOQ63_RS07550	Transposase	1,689,112	1,689,999
BOQ63_RS07585	Transposase	1,695,301	1,695,435
BOQ63_RS07615	IS5/IS1182 family transposase	1,699,532	1,699,984
BOQ63_RS07620**	IS5/IS1182 family transposase	1,699,981	1,700,334
BOQ63_RS07635	Transposase	1,704,820	1,706,307
BOQ63_RS07640	TnsA-like heteromeric transposase	1,706,304	1,706,978

*Transposase found upstream KVP1 right terminus present in bald strain B3.1

**Homologues of the transposase encoded by BOQ63_RS37135

## Discussion

Actinobacterial genomes readily undergo rearrangements, whereby more than 1 Mb of genomic DNA can be lost (Hoff et al. 2018; Redenbach et al. 1993; Zhang et al. 2020). Here, we provide evidence that protoplast formation and regeneration in *K. viridifaciens* can lead to profound genomic rearrangements in the chromosome as well as loss of (large parts of) the megaplasmid KVP1. Given that these genomic rearrangements translate into major phenotypic variations, caution should be taken when using protoplasts for creating mutants, in particular when using strains that carry natural plasmids.

Filamentous Actinobacteria grow by tip extension and develop multinucleated mycelia. Little is known on how these bacteria regulate the abundance and spatial distribution of chromosomes and extrachromosomal plasmids within the mycelium. Maintaining large plasmids such as KVP1 is costly, given that such elements can comprise about a fifth of the entire genome. Megaplasmids are often reservoirs for biosynthetic gene clusters, and their interactions with the chromosome have been suggested to be a driving force for horizontal gene transfer (Kinashi 2011). Loss of such plasmids not only affects morphological development but may also influence the production of specialised metabolites whose gene clusters are contained on the chromosome. In *Streptomyces hygroscopicus* elimination of pSHJG1 increased the production of validamycin A (Lu et al. 2017), while holomycin yield was boosted when pSCL4 was lost in *Streptomyces clavuligerus* (Álvarez-Álvarez et al. 2014; Charusanti et al. 2012). Whether the loss of KVP1 has a similar effect on production of specialised metabolites in *K. viridifaciens* remains to be elucidated.

**Fig. 5:**
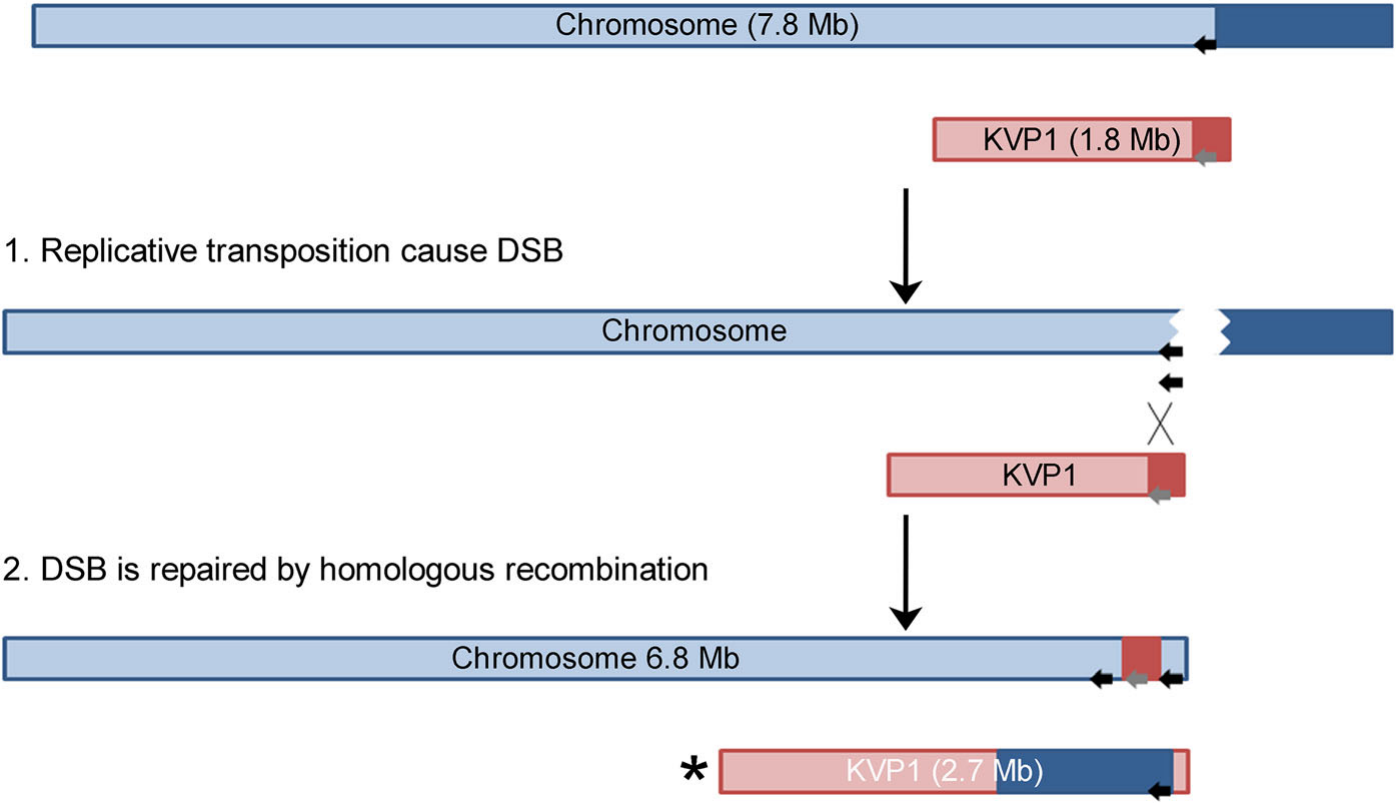
Proposed model for the genomic rearrangements and deletions identified in strain B3.1. Replicative transposition of a transposable element (black arrow) located on the chromosome creates a double-stranded break (DSB). Subsequent repair of the DSB by homologous recombination between the chromosome and KVP1 plasmid (carrying numerous transposable elements), leads to an exchange of the right arms of both replicons and changes in their size. Please note that the larger KVP1 megaplasmid variant is lost in strain B3.1

We observed that close to one fourth (23%) of the colonies derived from regenerated protoplasts of *K. viridifaciens* were defective in aerial growth and sporulation. Similar morphological defects have been described for *S. clavuligerus*, *Streptomyces lividans* and *Streptomyces coelicolor* upon loss of their plasmids (Álvarez-Álvarez et al. 2014; Hsu and Chen 2010). While the loss of KVP1 in *K. viridifaciens* may explain the arrest in morphological development, we here show that such plasmid-lacking derivatives can also carry other profound deletions in the chromosome, which could equally well contribute to this phenotype. By sequencing one revertant that had lost KVP1 we found that this strain had also lost approximately 1.5 Mb of the right arm of the chromosome. Such genetic instability is typical of streptomycetes, and can affect morphological differentiation, but also phenotypic traits associated with natural products, such as pigmentation, antibiotic biosynthesis and antibiotic resistance (Leblond and Decaris 1994; Zhang et al. 2020). Transposable elements were suggested as the principal cause of genetic instability (Lin and Chen 1997). The loss of KVP1 and the chromosomal deletions in the right arm could be the consequence of replicative transposition between the chromosome and the megaplasmid (Fig. 5). Notably, while most KVP1-located sequences were absent in the sequenced strain, including those required for autonomous replication of this megaplasmid, we identified a high coverage of sequences originally located at the end of the right arm of KVP1. This could be explained by an exchange between the right arms of KVP1 and the chromosome. A replicative transposition event between two linear replicons often results in the loss of chromosomal terminal regions and recombination of transposable elements (Chen et al. 2002). The presence of IS elements located at the chromosomal and plasmid termini could provide the basis for homologous recombination between these DNA molecules. It has been previously shown that homologous copies of IS elements could serve as substrates for the recombination machinery, creating chromosomal rearrangements in the genomes of *Lactococcus lactis* and *Escherichia coli* (Daveran-Mingot et al. 1998; Gaffé et al. 2011). In the case of colony B3.1 that had arisen from protoplast regeneration, our results suggest that a possible cause of the genomic rearrangement was the replicative transposition of an IS, exerted by the BOQ63_RS37135 transposase (black arrow in Fig. 5b). Following replicative transposition, a double-stranded break occurs at the site of transposon excision. This break is repaired by recombination with homologous genes located on IS elements present in the right arm of KVP1 (shown as a grey arrow in the KVP1 right arm), which are abundantly present (see Table 4, genes highlighted with **). This recombination might force the interchange of terminal arms. This hypothetical model would explain the genome size reduction of B3.1 (6,787,546 bp).

We previously identified several genes required for plasmid replication and segregation on KVP1 (Ramijan et al. 2017). Interestingly, bioinformatics analysis indicated that the conserved *tpg* gene, which encodes the terminal protein required for capping of the telomeres and thereby contributing to its protection, is absent in *K. viridifaciens*. Instead, we identified homologues of the Tac-Tpc system, encoded by the adjacent BOQ63_42425 and BOQ63_42430 genes, respectively. The *tac/tpc* genes were first identified in the linear *Streptomyces* plasmid SCP1 (Huang et al. 2007). In *K. viridifaciens*, the *tac*-*tpc* genes are located on the right arm of the chromosome and no copies are found on KVP1. This implies that the encoded proteins are required for maintenance of both the chromosome and plasmid telomeres. The use of the Tac-Tpc system by *K. viridifaciens* also indicates that, like in streptomycetes, different systems are used for maintenance of telomeres in *Kitasatospora* species, given that *Kitasatospora setae* contains the *tpg* gene (Ichikawa et al. 2010).

The frequency of aberrant phenotypes after protoplast regeneration is higher than the phenotypic heterogeneity obtained after outgrowth of spores, which typically is in the order of 1% (Gravius et al. 1993; Hoff et al. 2018; Leblond et al. 1989; Lin and Chen 1997; Redenbach et al. 1993; Zhang et al. 2020). An explanation for the high frequency of aberrant mutants in colonies arising after protoplasting may relate to the activation of transposable elements contained in the terminal regions of the chromosome and/or the KVP1 plasmid. The activation of transposases are typically stimulated by stressful conditions, such as radiation, oxidative stress, temperature or inhibitory concentrations of metals and antibiotics (Vandecraen et al. 2017). It was recently demonstrated that elevated levels of osmolytes induces hyperosmotic stress (Fuchino et al. 2017; Ramijan et al. 2018), which are conditions that are also used during preparation of protoplasts. This stress could also stimulate transposition events and consequent chromosomal rearrangements. Consistent with this idea is that other cell wall-deficient cells, called L-forms, which have likewise been exposed to osmotic stress conditions, carry chromosomal deletions. In this context it is interesting to note that in three independent L-form lineages of *K. viridifaciens* deletions in the right chromosomal arms were found in addition to loss of KVP1 (Ramijan et al. 2018). These three strains retained a similar region of KVP1 in their genomes, with a size of 164,773 bp for *alpha* and M1, and 164,642 bp for M2. These are very similar to the KVP1-sequences remaining (164,769 bp) in the bald protoplast regenerant B3.1 in terms of length and content. Notably, in strain B3.1 as well as the three independent L-form mutants the BOQ63_42425 and BOQ63_42430 genes are still found. Given that these genes are likely involved in telomere maintenance (see above), their presence could suggest that the chromosomes remain linear after these dramatic recombination steps. However, this awaits further experimental evidence.

Chromosomal rearrangements are often detrimental for the fitness of a unicellular organism. However, it was recently shown that in *Streptomyces* chromosomal rearrangements may increase the diversity and production of specialized metabolites, including antibiotics (Zhang et al. 2020). A division of labour strategy would allow a colony to have a mixture of mutant and wild-type chromosomes, where the mutant cells are virtually sterile and become specialized in the production of antibiotics, while the cells containing wild-type chromosomes are efficient spore producers (Zhang et al. 2020). Thus, while some genetic variation may naturally exist within the mycelium, we expect that exposure to high levels of osmolytes, associated with growth and subsequent protoplast formation, generates stress and dramatically increases chromosomal changes. This study provides a starting point to further characterise these changes and to investigate their consequences, which may lead to exciting new insights into the biology of these prolific antibiotic producers.

## Electronic supplementary material

Below is the link to the electronic supplementary material.

Supplementary material 1 (PDF 1414 kb)

## References

[CR1] Abrudan MI, Smakman F, Grimbergen AJ, Westhoff S, Miller EL, van Wezel GP, Rozen DE (2015). Socially mediated induction and suppression of antibiosis during bacterial coexistence. Proc Natl Acad Sci USA.

[CR2] Álvarez-Álvarez R (2014). A 1.8-Mb-reduced *Streptomyces clavuligerus* genome: relevance for secondary metabolism and differentiation. Appl Microbiol Biotechnol.

[CR3] Barka EA (2016). Taxonomy, physiology, and natural products of *Actinobacteria*. Microbiol Mol Biol Rev.

[CR4] Bentley SD (2002). Complete genome sequence of the model actinomycete *Streptomyces coelicolor* A3(2). Nature.

[CR5] Blin K (2019). antiSMASH 5.0: updates to the secondary metabolite genome mining pipeline. Nucl Acids Res.

[CR6] Charusanti P (2012). Exploiting adaptive laboratory evolution of *Streptomyces clavuligerus* for antibiotic discovery and overproduction. PLoS ONE.

[CR7] Chater KF (2001). Regulation of sporulation in *Streptomyces coelicolor* A3(2): a checkpoint multiplex?. Curr Opin Microbiol.

[CR8] Chater KF, Kinashi H, Meinhardt F, Klassen R (2007). Streptomyces linear plasmids: their discovery, functions, interactions with other replicons, and evolutionary significance. Microbial linear plasmids.

[CR9] Chen CW (1996). Complications and implications of linear bacterial chromosomes. Trends Genet.

[CR10] Chen CW, Huang CH, Lee HH, Tsai HH, Kirby R (2002). Once the circle has been broken: dynamics and evolution of *Streptomyces* chromosomes. Trends Genet.

[CR11] Claessen D, de Jong W, Dijkhuizen L, Wösten HAB (2006). Regulation of *Streptomyces* development: reach for the sky!. Trends Microbiol.

[CR12] Claessen D, Rozen DE, Kuipers OP, Søgaard-Andersen L, van Wezel GP (2014). Bacterial solutions to multicellularity: a tale of biofilms, filaments and fruiting bodies. Nat Rev Microbiol.

[CR13] Daveran-Mingot ML, Campo N, Ritzenthaler P, Le Bourgeois P (1998). A natural large chromosomal inversion in *Lactococcus lactis* is mediated by homologous recombination between two insertion sequences. J Bacteriol.

[CR14] Fuchino K, Flärdh K, Dyson P, Ausmees N (2017). Cell-biological studies of osmotic shock response in *Streptomyces* spp.. J Bacteriol.

[CR15] Gaffé J, McKenzie C, Maharjan RP, Coursange E, Ferenci T, Schneider D (2011). Insertion sequence-driven evolution of *Escherichia coli* in chemostats. J Mol Evol.

[CR16] Girard G, Willemse J, Zhu H, Claessen D, Bukarasam K, Goodfellow M, van Wezel GP (2014). Analysis of novel *kitasatosporae* reveals significant evolutionary changes in conserved developmental genes between *Kitasatospora* and *Streptomyces*. Antonie Van Leeuwenhoek.

[CR17] Gravius B, Bezmalinović T, Hranueli D, Cullum J (1993). Genetic instability and strain degeneration in *Streptomyces rimosus*. Appl Environ Microbiol.

[CR18] Hoff G, Bertrand C, Piotrowski E, Thibessard A, Leblond P (2018). Genome plasticity is governed by double strand break DNA repair in *Streptomyces*. Sci Rep.

[CR19] Hopwood DA (2006). Soil to genomics: the *Streptomyces* chromosome. Annu Rev Genet.

[CR20] Hsu CC, Chen CW (2010). Linear plasmid SLP2 is maintained by partitioning, intrahyphal spread, and conjugal transfer in *Streptomyces*. J Bacteriol.

[CR21] Huang CH (2007). The telomere system of the *Streptomyces* linear plasmid SCP1 represents a novel class. Mol Microbiol.

[CR22] Ichikawa N (2010). Genome sequence of *Kitasatospora setae* NBRC 14216T: an evolutionary snapshot of the family *Streptomycetaceae*. DNA Res.

[CR23] Kieser T, Bibb MJ, Buttner MJ, Chater KF, Hopwood DA (2000). Practical *Streptomyces* genetics.

[CR24] Kinashi H (2011). Giant linear plasmids in *Streptomyces*: a treasure trove of antibiotic biosynthetic clusters. J Antibiot Tokyo.

[CR25] Kirby R, Chen CW, Dyson P (2011). Genome architecture. Streptomyces: molecular biology and biotechnology.

[CR26] Kronheim S (2018). A chemical defence against phage infection. Nature.

[CR27] Leblond P, Decaris B (1994). New insights into the genetic instability of *Streptomyces*. FEMS Microbiol Lett.

[CR28] Leblond P, Demuyter P, Moutier L, Laakel M, Decaris B, Simonet JM (1989). Hypervariability, a new phenomenon of genetic instability, related to DNA amplification in *Streptomyces ambofaciens*. J Bacteriol.

[CR29] Lin YS, Chen CW (1997). Instability of artificially circularized chromosomes of *Streptomyces lividans*. Mol Microbiol.

[CR30] Lu C, Wu H, Su X, Bai L (2017). Elimination of indigenous linear plasmids in *Streptomyces hygroscopicus* var. *jinggangensis* and *Streptomyces* sp. FR008 to increase validamycin A and candicidin productivities. Appl Microbiol Biotechnol.

[CR31] Medema MH (2010). The sequence of a 1.8-mb bacterial linear plasmid reveals a rich evolutionary reservoir of secondary metabolic pathways. Genome Biol Evol.

[CR32] Mochizuki S, Hiratsu K, Suwa M, Ishii T, Sugino F, Yamada K, Kinashi H (2003). The large linear plasmid pSLA2-L of *Streptomyces rochei* has an unusually condensed gene organization for secondary metabolism. Mol Microbiol.

[CR33] Nindita Y (2015). The *tap*-*tpg* gene pair on the linear plasmid functions to maintain a linear topology of the chromosome in *Streptomyces rochei*. Mol Microbiol.

[CR34] Ramijan K, van Wezel GP, Claessen D (2017). Genome sequence of the filamentous actinomycete *Kitasatospora viridifaciens*. Genome Announc.

[CR35] Ramijan K (2018). Stress-induced formation of cell wall-deficient cells in filamentous actinomycetes. Nat Commun.

[CR36] Redenbach M (1993). The *Streptomyces lividans* 66 chromosome contains a 1 Mb deletogenic region flanked by two amplifiable regions. Mol Gen Genet.

[CR37] Stuttard C (1982). Temperate phages of *Streptomyces venezuelae*: lysogeny and host specificity shown by phages SV1 and SV2. J Gen Microbiol.

[CR38] van der Heul HU, Bilyk BL, McDowall KJ, Seipke RF, van Wezel GP (2018). Regulation of antibiotic production in Actinobacteria: new perspectives from the post-genomic era. Nat Prod Rep.

[CR39] Vandecraen J, Chandler M, Aertsen A, Van Houdt R (2017). The impact of insertion sequences on bacterial genome plasticity and adaptability. Crit Rev Microbiol.

[CR40] Zhang Z (2020). Antibiotic production in *Streptomyces* is organized by a division of labour through terminal genomic differentiation. Sci Adv.

