# Supplementary material for: Genome rearrangements and megaplasmid loss in the filamentous bacterium Kitasatospora viridifaciens are associated with protoplast formation and regeneration
Source: Antonie Van Leeuwenhoek. 2020 Feb 14;113(6):825–37. doi: 10.1007/s10482-020-01393-7 (PMC7188733; doi:10.1007/s10482-020-01393-7)
Supplement: Supplementary file 1 — Supplementary material 1 (PDF 1414 kb) [file 10482_2020_1393_MOESM1_ESM.pdf]

Figure S1

A

Region	Type	From	To	Most similar known cluster		Similarity	MIBiG BGC-ID
The following regions are from record MPLE01000001.1 (Streptomyces viridifaciens):							

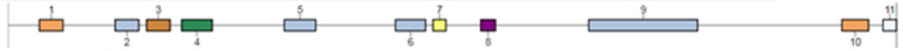

Region	Type	From	To	Most similar known cluster		Similarity	MIBiG BGC-ID
Region 1	T3pks	55019	96101	Actinomycin	NRPS	10%	BGC0000296
Region 2	Lanthipeptide - Nrpsfragment	186747	228158	Viomycin	NRPS	9%	BGC0000458
Region 3	Arylpolyene	241651	282784	WS9326	NRPS	5%	BGC0001297
Region 4	Nrps	302617	356025				
Region 5	Nrps - Nucleoside	481446	536185	Tunicamycin	other	42%	BGC0000880
Region 6	T1pks - Nrps	675210	727626	Oxazolomycin	hybrid	15%	BGC0001106
Region 7	Lanthipeptide	740507	763068				
Region 8	Terpene	823523	849476	Carotenoid	terpene	45%	BGC0000633
Region 9	Nrps - T1pks	1011856	1201449	Candicidin	polyketide	76%	BGC0000034
Region 10	T1pks	1452695	1499210				
Region 11	Blactam	1524280	1547868	Clavams	other	64%	BGC0001151

B

Region	Type	From	To	Most similar known cluster		Similarity	MIBiG BGC-ID
The following regions are from record MPLE01000004.1 (Streptomyces viridifaciens):							

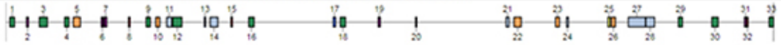

Region	Type	From	To	Most similar known cluster		Similarity	MIBiG BGC-ID
Region 1	Nrps	43540	90359	Capreomycin	NRPS	9%	BGC0000316
Region 2	Terpene	211185	230997				
Region 3	Nrps	340765	421797	Lipopeptide 8D1-1 / lipopeptide 8D1-2	NRPS	15%	BGC0001370
Region 4	Nrpsfragment	596569	638812	Meilingmycin	polyketide	4%	BGC0000093
Region 5	T2pks	683975	754674	Spore pigment	polyketide	83%	BGC0000271
Region 6	Terpene	966842	986462	Acarviostatin	saccharide	11%	BGC0000804
Region 7	Terpene	996986	1021419	Hopene	terpene	61%	BGC0000663
Region 8	Siderophore	1241877	1255028				
Region 9	Nrpsfragment	1418560	1460286				
Region 10	Otherks	1515975	1556086	Kijanimicin	polyketide	4%	BGC0000082
Region 11	Nrpsfragment - T1pks	1626509	1677101	Vancomycin	NRPS	17%	BGC0000455
Region 12	Nrps	1684526	1779221	Friulimicin	NRPS	24%	BGC0000354
Region 13	Bacteriocin	2007281	2017303				
Region 14	Nrps - Betalactone	2064569	2142082	Lomaiviticin	polyketide	6%	BGC0000240
Region 15	Siderophore	2274617	2287884				
Region 16	Nrps	2453955	2507665	Teleocidin B	hybrid	50%	BGC0001085
Region 17	Lap	3307340	3330204				
Region 18	Nrps	3378027	3430494	Skyllamycin	NRPS	20%	BGC0000429
Region 19	Terpene	3760799	3781772	Geosmin	other	100%	BGC0001181
Region 20	Butyrolactone	4138981	4149459	Pristinamycin	hybrid	3%	BGC0000952
Region 21	Thiopeptide - Lap	5051227	5080751				
Region 22	T2pks	5131444	5203500	Chlortetracycline	polyketide	88%	BGC0000209
Region 23	T3pks	5550533	5589534	Herboxidiene	polyketide	2%	BGC0001065
Region 24	Bacteriocin - Butyrolactone	5660652	5680142	Methylenomycin	other	28%	BGC0000914
Region 25	Lanthipeptide	6078936	6101773	A54145	NRPS	5%	BGC0000291
Region 26	T3pks	6112996	6154051	Naringenin	terpene	100%	BGC0001310
Region 27	Nrps - T1pks	6284704	6457666	Gobichelin	NRPS	27%	BGC0000366
Region 28	Nrps - Otherks	6461811	6548854				
Region 29	Nrpsfragment	6790211	6831191				
Region 30	Nrps	7128111	7192080	Cyclomarin	NRPS	8%	BGC0000333
Region 31	Terpene	7457995	7479215	2-methylisoborneol	terpene	100%	BGC0000658
Region 32	Siderophore	7480413	7493514				
Region 33	Nrpsfragment	7706433	7750347	Herboxidiene	polyketide	3%	BGC0001065

Figure S1. AntiSMASH 5.0 output revealing the biosynthetic gene clusters contained on the megaplasmid (top) and chromosome (bottom) of *K. viridifaciens*. The biosynthetic gene clusters are numbered according to their localization on the replicon.

Figure S2

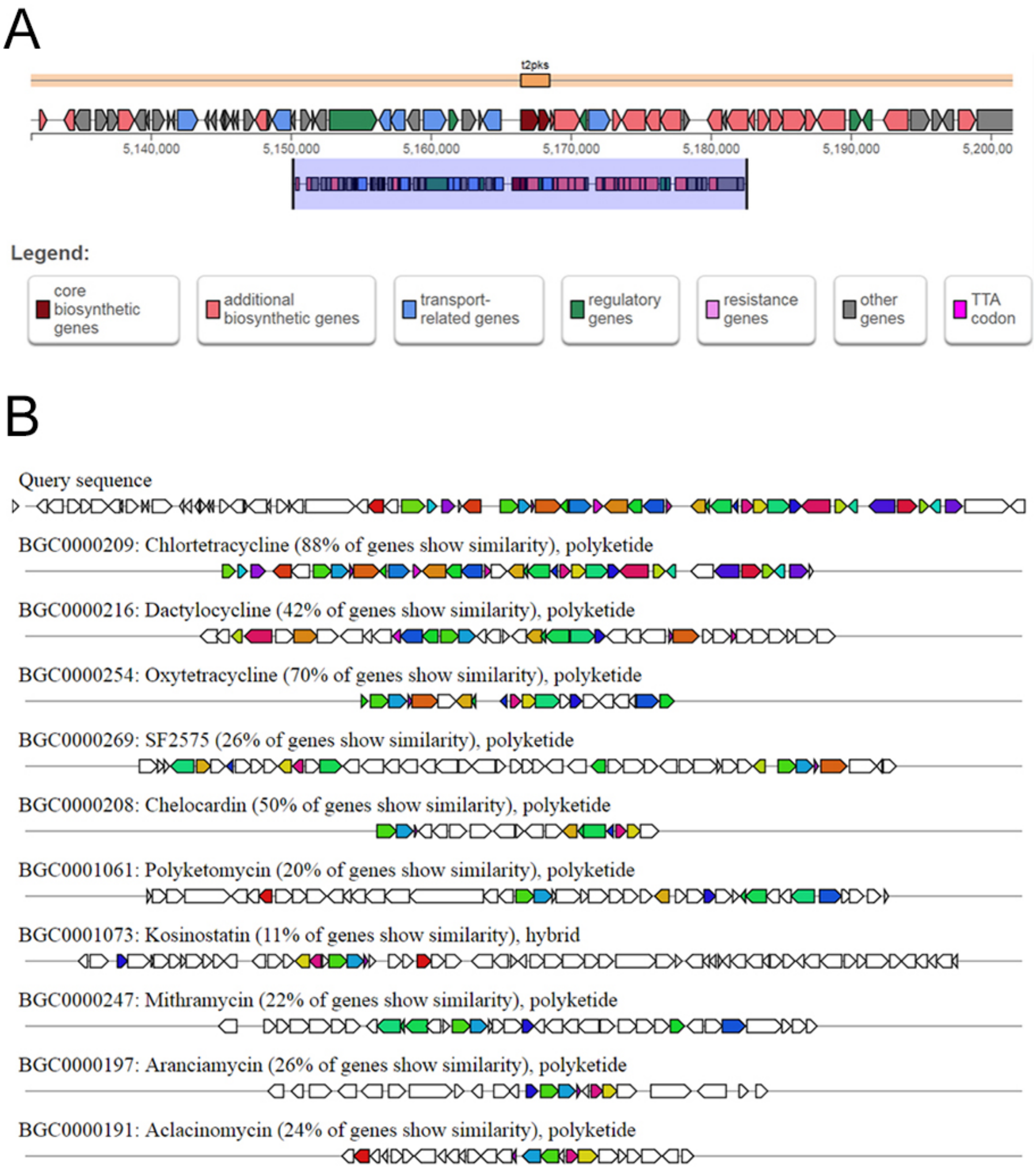

Figure S2. AntiSMASH 5.0 homology search of the *K. viridifaciens* tetracycline biosynthetic gene cluster. (A) Localization of the putative chlorotetracycline BGC in the chromosome of *K. viridifaciens*. (B) Comparison of the *K. viridifaciens* tetracyclin biosynthetic gene cluster with known tetracycline gene clusters from *Streptomyces aureofaciens* (BGC0000209), *Dactylosporangium sp.* SC14051 (BGC0000216), *Streptomyces rimosus* (BGC0000254), *Streptomyces sp*. SF2575 (BGC0000269), *Amycolatopsis sulphurea* (BGC0000208), *Streptomyces diastatochromogenes* (BGC0001061), *Micromonospora sp*. TP-A0468 (BGC0001073), *Streptomyces argillaceus* (BGC0000247), *Streptomyces echinatus* (BGC0000197) and *Streptomyces galilaeus* (BGC0000191).

Figure S3

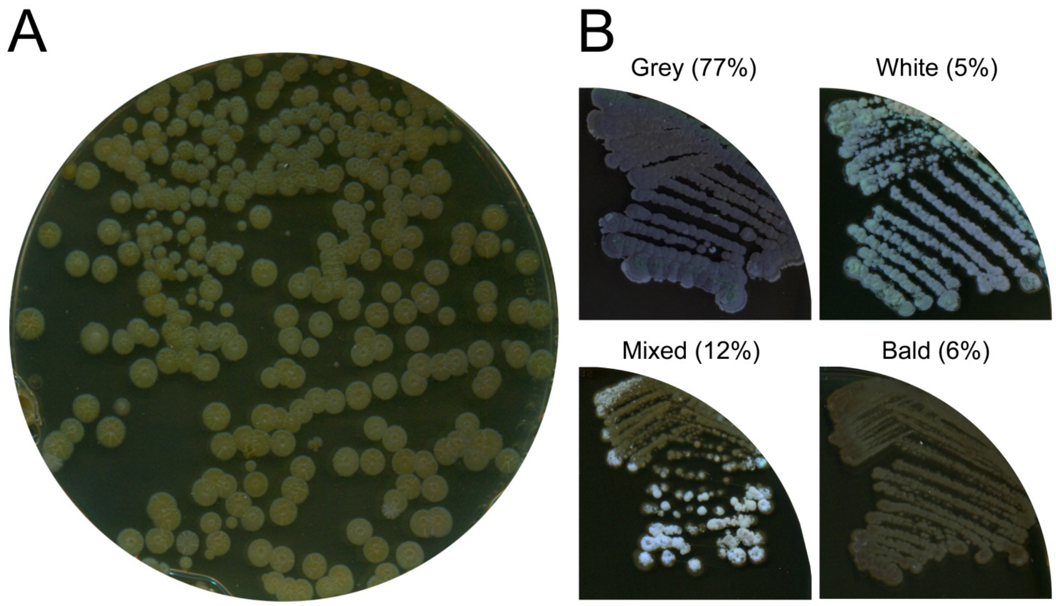

Figure S3. Protoplast regeneration generates morphological diversity in osmotically balanced medium. (A) Protoplasts regenerated on R5 medium yielded colonies that are unable to sporulate due to the high sucrose levels. (B) Subculturing of 149 randomly-picked colonies on MYM medium revealed dramatic developmental defects in 23% of the colonies. Whereas 77% of the colonies were able to form grey-pigmented sporulating colonies (similar to the wild-type), 5% of the colonies were white, 6% were bald, while 12% had a mixed appearance.
